# Exploratory Investigation of Intestinal Function and Bacterial Translocation After Focal Cerebral Ischemia in the Mouse

**DOI:** 10.3389/fneur.2018.00937

**Published:** 2018-11-19

**Authors:** Naoki Oyama, Katarzyna Winek, Priscilla Bäcker-Koduah, Tian Zhang, Claudia Dames, Martina Werich, Olivia Kershaw, Christian Meisel, Andreas Meisel, Ulrich Dirnagl

**Affiliations:** ^1^Department of Experimental Neurology, Charité – Universitätsmedizin, Corporate Member of Freie Universität Berlin, Humboldt Universitäts zu Berlin and Berlin Institute of Health, Berlin, Germany; ^2^Center for Stroke Research Berlin, Charité – Universitätsmedizin, Corporate Member of Freie Universität Berlin, Humboldt Universitäts zu Berlin and Berlin Institute of Health, Berlin, Germany; ^3^Neurocure Cluster of Excellence, Charité – Universitätsmedizin, Corporate Member of Freie Universität Berlin, Humboldt Universitäts zu Berlin and Berlin Institute of Health, Berlin, Germany; ^4^Institute for Medical Immunology, Charité – Universitätsmedizin, Corporate Member of Freie Universität Berlin, Humboldt Universitäts zu Berlin and Berlin Institute of Health, Berlin, Germany; ^5^Medical Department, Division of Hepatology and Gastroenterology, Charité – Universitätsmedizin, Corporate Member of Freie Universität Berlin, Humboldt Universitäts zu Berlin and Berlin Institute of Health, Berlin, Germany; ^6^Institute of Veterinary Pathology, Faculty of Veterinary Medicine, Freie Universität Berlin, Berlin, Germany; ^7^Department of Neurology, Charité – Universitätsmedizin, Corporate Member of Freie Universität Berlin, Humboldt Universitäts zu Berlin and Berlin Institute of Health, Berlin, Germany; ^8^German Center for Neurodegenerative Diseases (DZNE), Partner Site Berlin, Berlin, Germany; ^9^QUEST – Center for Transforming Biomedical Research, Berlin Institute of Health, Berlin, Germany

**Keywords:** bacterial translocation, gut-brain axis, gut permeability, gut associated lymphatic tissue, immune system, stroke, tight junctions

## Abstract

**Background and Purpose:** The gut communicates with the brain bidirectionally via neural, humoral and immune pathways. All these pathways are affected by acute brain lesions, such as stroke. Brain-gut communication may therefore impact on the overall outcome after CNS-injury. Until now, contradictory reports on intestinal function and translocation of gut bacteria after experimental stroke have been published. Accordingly, we aimed to specifically investigate the effects of transient focal cerebral ischemia on intestinal permeability, gut associated lymphoid tissue and bacterial translocation in an exploratory study using a well-characterized murine stroke model.

**Methods:** After 60 min of middle cerebral artery occlusion (MCAO) we assessed intestinal morphology (time points after surgery day 0, 3, 5, 14, 21) and tight junction protein expression (occludin and claudin-1 at day 1 and 3) in 12-week-old male C57Bl/6J mice. Lactulose/mannitol/sucralose test was performed to assess intestinal permeability 24–72 h after surgery. To investigate the influence of cerebral ischemia on the local immune system of the gut, main immune cell populations in Peyer's patches (PP) were quantified by flow cytometry. Finally, we evaluated bacterial translocation to extraintestinal organs 24 and 72 h after MCAO by microbiological culture and fluorescence *in situ* hybridization targeting bacterial 16S rRNA.

**Results:** Transient MCAO decreased claudin-1 expression in the ileum but not in the colon. Intestinal morphology (assessed by light microscopy) and permeability did not change measurably after MCAO. After MCAO, animals had significantly fewer B cells in PP compared to naïve mice.

**Conclusions:** In a murine model of stroke, which leads to large brain infarctions in the middle cerebral artery territory, we did not find evidence for overt alterations neither in gut morphology, barrier proteins and permeability nor presence of intestinal bacterial translocation.

## Introduction

Every year around 17 million people worldwide suffer their first stroke. Stroke patients are highly susceptible to medical complications, which influence outcome and increase the length of hospital stay, economic costs of treatment and burden of care given (1). Above all, infections are the most common post-stroke complication, associated with worsened outcome (1, 2). Dysphagia and systemic immunosuppression are the two most important factors in the pathogenesis of stroke-associated infections and predict the risk of post-stroke pneumonia independently (3). Additionally, it is well known that stroke patients suffer from a number of gastrointestinal complications, like gastrointestinal dysmotility or gastrointestinal hemorrhage (4). Importantly, the gut is a complex organ responsible not only for nutrient absorption; it is hormonally active and possesses its own immune and nervous systems. Since stroke leads to acute overactivation of the sympathetic nervous system and the HPA-axis, and consequently systemic immunosuppression (5), it has been hypothesized that gut permeability and motility may be affected after cerebral ischemia (5–7). Furthermore, recent evidence suggests that the population of commensal gut bacteria is profoundly disturbed after stroke. This may affect outcome after brain injury, mainly by interaction with the host immune system (8–10). Moreover, translocation of microbial components or of bacterial cells from the intestine has been proposed as a modulator of immune responses (11) or even as a source of systemic infections after stroke (12).

There is little and contradictory experimental or clinical evidence regarding post-stroke gut function. For instance, bacterial translocation after cerebral ischemia has been reported by some groups (12–15) but others did not observe this phenomenon (9). Therefore, the specific aim of the present study was to explore the influence of focal cerebral ischemia on (1) intestinal morphology and barrier function including tight junctions, (2) local immune system in intestine, and (3) bacterial translocation from intestinal lumen to MLNs and/or other possible organs. We opted to perform our study in a widely used murine model of transient MCAO. A large portion of our current understanding of the pathophysiology of stroke, including the effects of stroke on peripheral immunity, was derived from this model. To maximize putative effects of the brain lesion on gut function and morphology we chose an occlusion time of the MCA which produces large brain infarctions, comparable to so called “malignant” ischemic stroke of the middle cerebral artery territory in humans. Our provisional hypothesis was that severe brain infarction in murine stroke leads to early alterations in intestinal barrier function and immunity as well as bacterial translocation.

## Materials and methods

### Animals and housing

Male 12-week-old C57Bl/6J mice (Janvier, France) were used for the experiments. Mice were housed in plastic cages containing chip bedding on a 12 h light/dark cycle (7 a.m./7 p.m light change) and provided free access to food (standard chow) and water. Animals were exposed to environmental enrichment, such as mouse tunnel, igloo and running wheel (Plexx B.V, Elst, Netherlands). All experiments were approved by the local authority - Landesamt für Gesundheit und Soziales, Berlin, Germany, and conducted in accordance with national and international regulations.

### Experimental design

Presented data are pooled from experiments conducted according to 3 setups. In the experiment 1 (Supplemental Figure 1), we performed a sham operation or 60 min of middle cerebral artery occlusion (MCAO) and sacrificed mice on day 1 or 3. To assess the influence of brain ischemia on intestinal barrier function, local immune system and bacterial translocation, we evaluated: tight junction proteins expression (claudin-1 and occludin) in ileum and colon by Western blot, immune cell populations in Peyer's patches using flow cytometry and the existence of microorganisms in extraintestinal organs by cultivation and fluorescence *in situ* hybridization (FISH). Experiments were performed with *n* = 20 for the 24 h time point (naïve/sham/MCAO *n* = 6/6/8) and *n* = 21 for the 72 h time point (naïve/sham/MCAO *n* = 6/7/8).

In the experiment 2 (Supplemental Figure 1), we investigated physiological intestinal permeability using lactulose/mannitol/sucralose test on day 2 or day 3. Following n numbers were used for this study: time point day 2 *n* = 15 (sham/MCAO *n* = 6/9), time point day 3 *n* = 25 (naïve/sham/MCAO *n* = 6/8/11). The aim of experiment 3 (Supplemental Figure 1) was investigation of the morphological changes in the intestine at different time points after sham- or MCAO surgery (day 0, 3, 5, 14, and 21) with *n* = 35 (day 0, sham *n* = 3, MCAO *n* = 3; day 3, sham *n* = 3, MCAO *n* = 3; day 5, sham *n* = 3, MCAO *n* = 4; day 14, sham *n* = 3, MCAO n = 5; day 21, sham *n* = 3, MCAO *n* = 5). Throughout the experiments, general health status was examined every day (16) and infarct volume was assessed using histology (experiment setup 1) or magnetic resonance imaging on day one after surgery (experiment setup 2 and 3) as quality control for successful MCAO.

### Methods to prevent bias, exclusion criteria, quality management

Animals were randomly assigned to the naïve, sham and MCAO groups using random numbers generator. Flow cytometry, Western blot, infarct volume analysis and histology investigations were performed by experimentators blinded for group assignment. Exclusion criteria: Animals of the MCAO group that did not show signs of infarction in histological/MRI investigation (*n* = 1), mice dying on the day of the surgery (*n* = 2) as well as animals with histologically detectable brain lesions in sham operated mice (*n* = 1) were excluded from the study. Moreover, animals that did not reach the experimental endpoint because of mortality/ reaching a humane endpoint during the experiments were excluded from the analysis (*n* = 13). Additionally, in the specific analyses samples were excluded only in case of technical problems. Detailed list of all excluded animals and experimental setups are provided in the Data Supplement.

All experiments were conducted in a laboratory environment with structured quality management (ISO9001-2008).

### Data analysis, statistics, and open data, ARRIVE guidelines

Our study was undertaken as an exploratory analysis of alterations of gut morphology, intestinal barrier and function, changes in gut associated lymphoid tissue, as well as potential bacterial translocation after experimental murine MCAO. The results of our study can stimulate hypothesis generation, and inform the determination of appropriate sample sizes for further investigation. Due to the explorative nature of our investigation, experimental protocols were not preregistered. Besides descriptive statistics, formal statistical testing of such exploratory data sets is not appropriate due to the lack of prespecified hypotheses and definition of a primary endpoint, inability to appropriately correct for testing of multiple comparisons, absence of a priori power calculation and low statistical power due to high biological variance and small group sizes (17), as well as the “garden of the forking paths problem” (18). Since it is nevertheless customary in the current biomedical literature to analyze such data sets with test statistics we have followed this practice as indicated. All original data reported in this study are made available via Figshare (https://doi.org/10.6084/m9.figshare.6727856)

Sample sizes were chosen according to previous experience with the model and the methods and the available literature, but not informed by formal sample size calculation due to the exploratory nature of this study. At conventional alpha and ß error levels (0.05, 0.2) it is reasonable to assume that most comparisons made only allow the detection of large effect sizes (*d* > 0.8).

Data from cultivation analyses are shown as the median and dot plots, all other data are expressed as the mean ± standard deviation and dot plots. Differences between two means were examined by the Mann-Whitney U test in permeability test (experiment 2–1, Supplemental Figure 3) and bacterial translocation data. Kruskal-Wallis test with Dunn's correction for multiple comparisons was used for multiple comparisons in Western blot, permeability test (experiment 2-2, Figure 3), immunological and bacterial translocation data. Data from the permeability tests were analyzed for significant outliers using the ROUT method (*Q* = 1%) (19). Statistics were performed using GraphPad Prism software version 6.00 (GraphPad Software, La Jolla, CA, USA). Detailed methods are available in the online Data Supplement.

Reporting of our results complies with the ARRIVE guidelines (https://www.nc3rs.org.uk/arrive-guidelines)

## Results

### Lack of evidence that 60 min focal cerebral ischemia in the mouse induces changes in intestinal morphology

**Figure 1:**
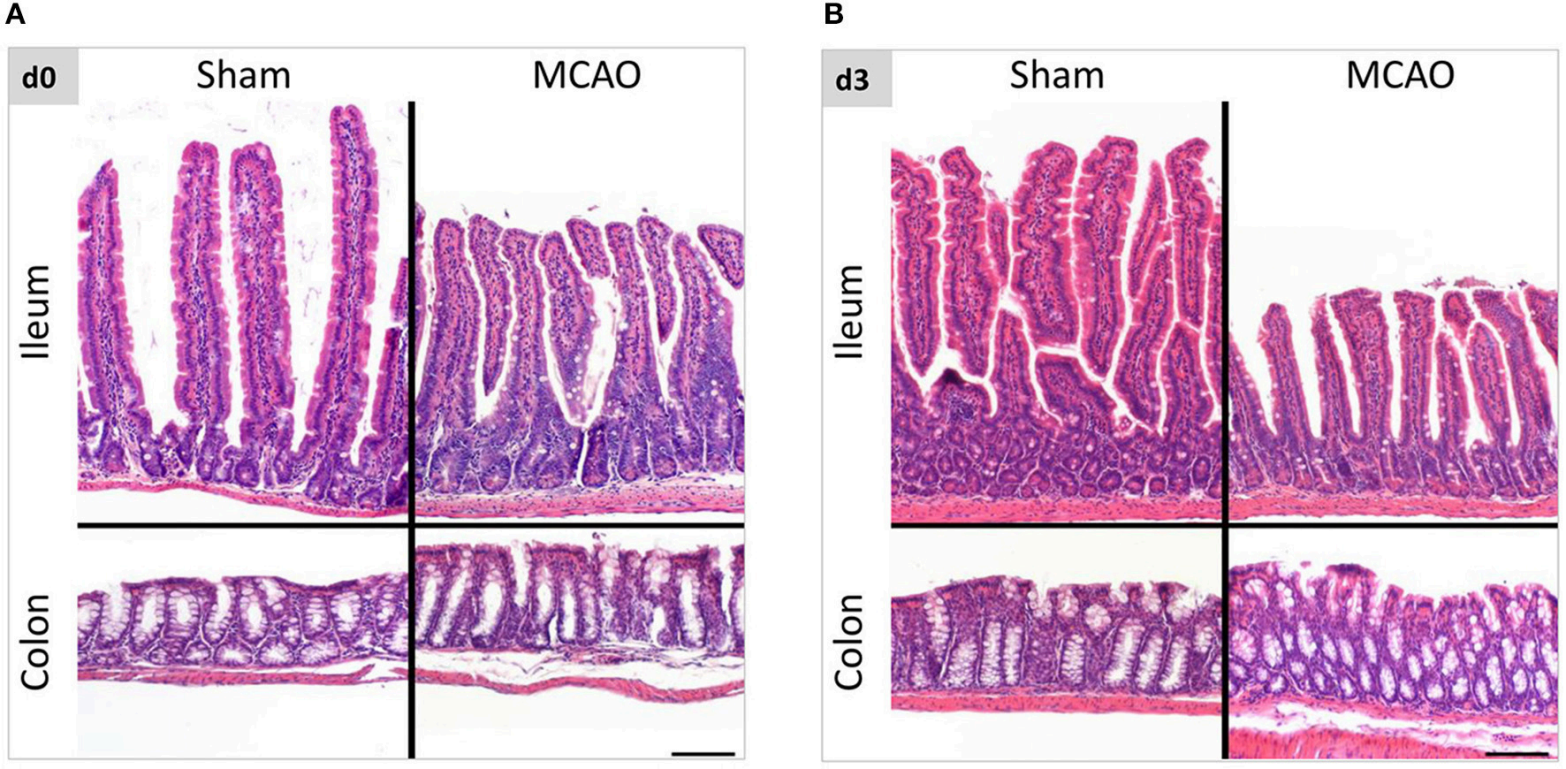
Intestinal morphology was not affected by ischemic brain injury. **(A)** Representative hematoxylin and eosin staining of ileum and colon in sham- and MCAO-operated mice on the day of the surgery and 3 days thereafter **(B)**. The variability in the villi length is a physiological phenomenon (decreasing aboral in the small intestine), depending on sectioning and unavoidable with the Swiss roll technique. Scale bar = 100 μm.

To evaluate the influence of cerebral ischemia on intestinal morphology, we examined histological changes in the entire intestine with hematoxylin & eosin staining. Within the first 3 weeks after cerebral ischemia (evaluation on the day of the surgery, day 3, day 5, 14, and 21 thereafter), we found no apparent evidence of disarrangement, erosion, ulceration and inflammation in intestinal submucosa and mucosa including epithelial cells and lamina propria. (Figures 1, Supplemental Figure 2). Presented pictures were taken in several anatomical locations of the intestine, hence differences between the length of the villi. This variability is a physiological phenomenon (villi length decreasing aboral in the small intestine), depending on sectioning and unavoidable with the Swiss roll technique (20).

### Alterations of claudin-1 expression in ileum after experimental stroke

**Figure 2:**
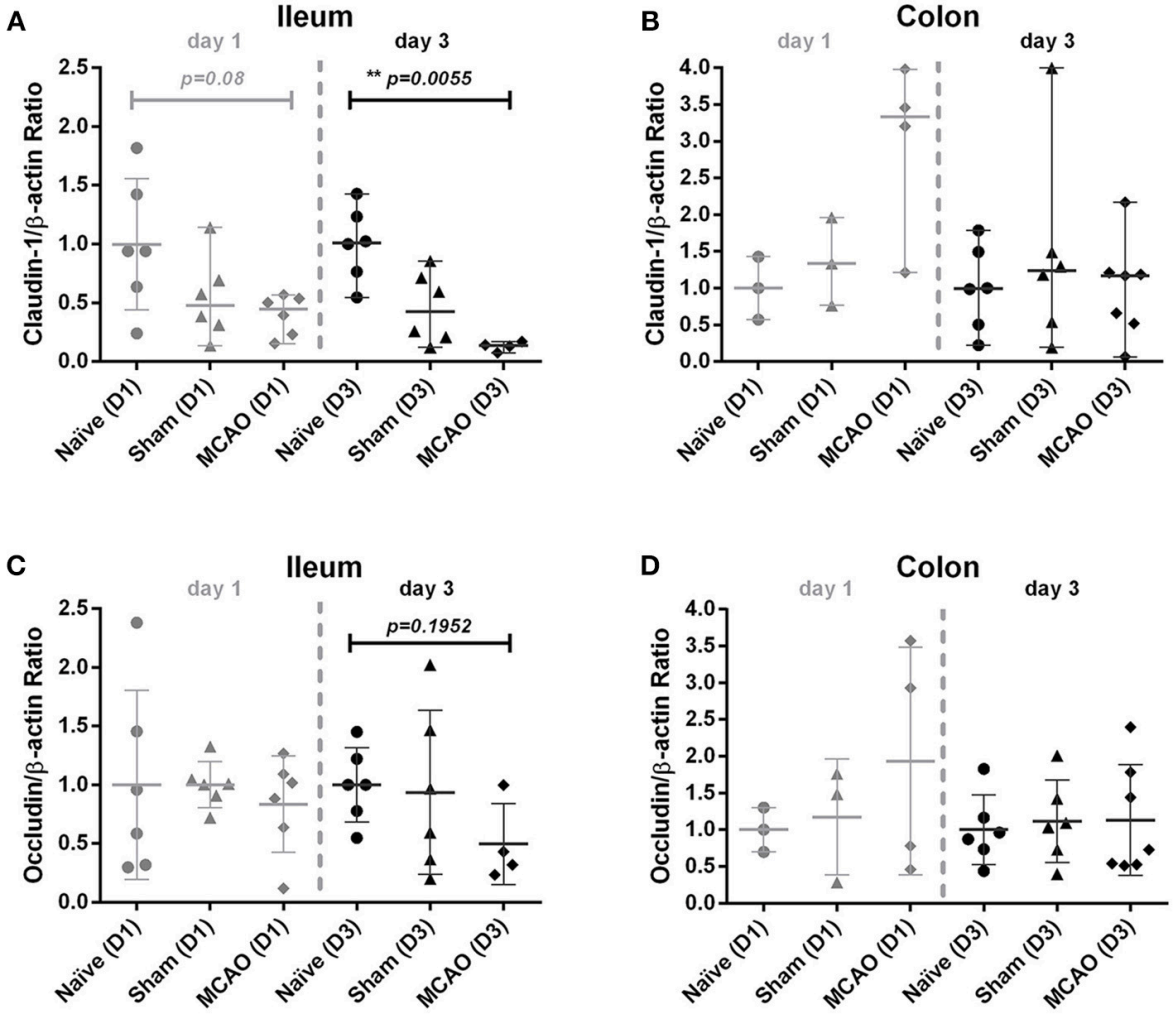
Ischemia decreased claudin-1 expression in the ileum, but not in the colon. Membranous proteins were extracted for Western blot analysis. **(A)** Western blot analysis of claudin-1 and β-actin in the ileum, **(B)** claudin-1 and β-actin in the colon, **(C)** occludin and β-actin in the ileum, and **(D)** occludin and β-actin in colon on day 1 and 3. All values were normalized by setting the densitometry of the naïve control sample to 1.0. Data are expressed as the mean ± standard deviation. The groups were compared using Kruskal-Wallis test with Dunn's *post-hoc*. MCAO indicates middle cerebral artery occlusion.

To evaluate intestinal barrier integrity in the first days after brain injury, we examined claudin-1 and occludin expression in the membrane fraction from ileum and colon homogenates 1 and 3 days after cerebral ischemia. As shown in Figure 2A, we observed a decrease in claudin-1 expression in sham and MCAO groups on day 1 and day 3 in the ileum (for MCAO compared to the naïve controls, *p* = 0.08 for day 1 and *p* = 0.00055 for day 3, Kruskal-Wallis test with Dunn's correction). In colon (analysis for day 1 was performed only in one sub experiment), no systematic changes in claudin-1 expression were apparent (Figure 2B). Occludin expression did not demonstrate systematic alterations in expression in ileum and colon samples from animals after MCAO (Figures 2C,D).

### Lack of evidence for altered intestinal permeability after MCAO when compared to naïve and sham operated animals

**Figure 3:**
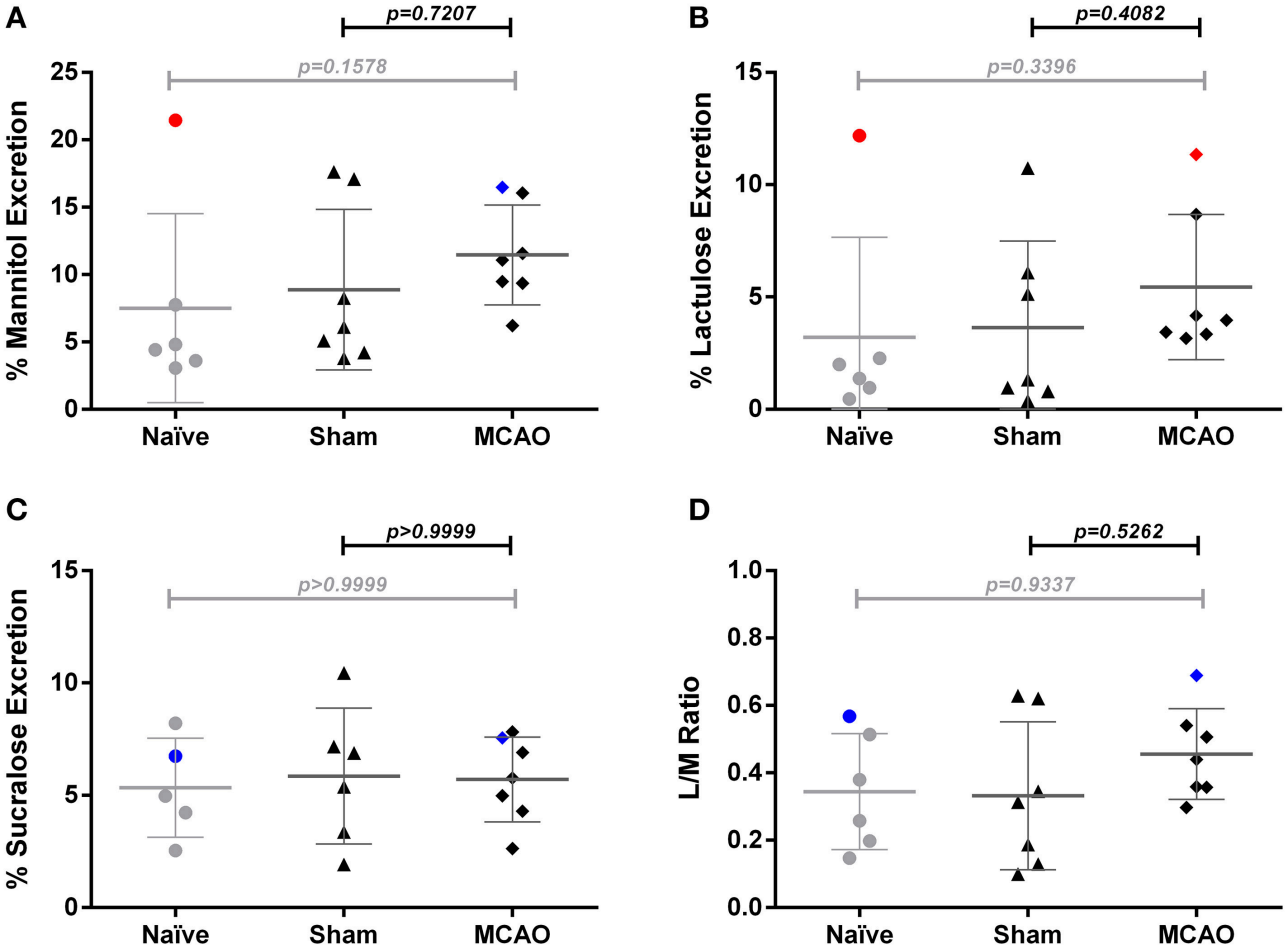
Sugar test revealed no significant changes of intestinal permeability 3 days after ischemia. **(A)** percent urinary excretion of mannitol, **(B)** lactulose, and **(C)** sucralose, **(D)** excretion ratio of lactulose and mannitol ratio (L/M ratio). Percent excretion in urine = 100 × urinary concentration of sugar (mg/mL) × urine volume (mL)/total ingested (mg). Outliers identified by the ROUT method are marked in red. In blue are depicted animals that were identified as outliers in previous tests (e.g., the MCAO animal is an outlier only for lactulose test but not mannitol, sucralose or lactulose/mannitol ratio). Data are expressed as the mean ± standard deviation. The groups were compared using Kruskal-Wallis test with Dunn's *post-hoc*. MCAO indicates middle cerebral artery occlusion.

To assess intestinal barrier function after cerebral ischemia, we performed mannitol/lactulose/sucralose absorption tests in mice subjected to MCAO or sham operation and naïve control groups. Compared to naïve and sham-operated mice, we observed no systemic alterations in lactulose and mannitol excretion on day 2–3 after MCAO (Figures 3A–D). Similarly, we did not observe any difference in lactulose/mannitol/sucralose excretion between sham and MCAO-operated groups at time point 1–2 days after surgery. (Supplemental Figures 3A–D). We noted high variability between individual mice within the groups. The naïve group in the measurement from day 2–3 after MCAO includes one significant outlier identified by the ROUT method (data point in red). Another outlier was found in the MCAO group from this time point in the lactulose test (data point in red). In blue, we depicted animals that were identified as outliers in previous sugar tests (e.g., the MCAO animal is an outlier only for lactulose test but not mannitol, sucralose or lactulose/mannitol ratio). Given the exploratory character of our experimental approach, as well as the lack of prespecified criteria for the exclusion of outliers, we did not exclude them from the analysis. The measurement on day 1–2 after surgery (Supplemental Figures 3A–D) did not contain any significant outliers.

### Transient middle cerebral artery occlusion reduces B cell counts in peyer's patches compared to naïve mice

**Figure 4:**
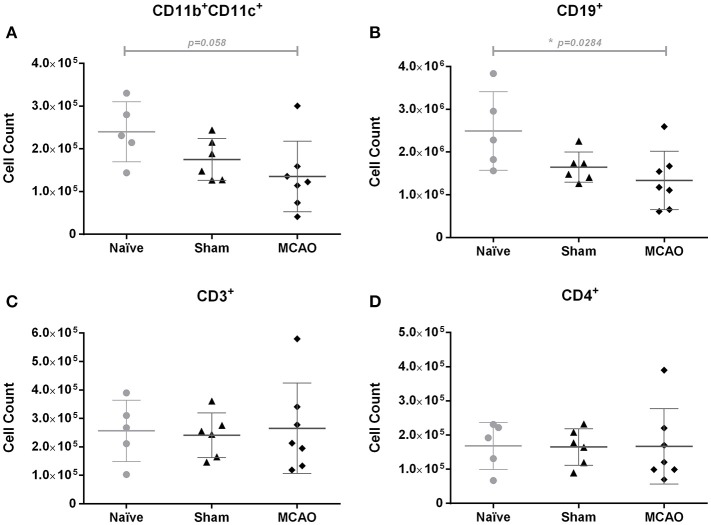
Ischemia induced a slight decrease of CD11b^+^CD11c^+^ cells and a significant reduction of CD19^+^ cells in Peyer's patches on day 1, when compared to the naïve group. Immune cell populations in Peyer's patches were analyzed by flow cytometry (FACS). **(A)** Absolute numbers of CD11b^+^CD11c^+^ cells (myeloid dendritic cells), **(B)** CD19^+^ cells (total B cells), **(C)** CD3^+^ cells (total T cells), and **(D)** CD4^+^ cells (helper T cells). Data are expressed as the mean ± standard deviation. The groups were compared using Kruskal-Wallis test with Dunn's *post-hoc*. *p ≤ 0.05 (naïve vs. MCAO).

In this explorative analysis, we focused on immune cell populations in Peyer's patches (PP). To elucidate whether stroke induces an early local immune depression state within the gut, we investigated immune cell populations in PP 1 day after surgery. Our analysis revealed decreased numbers of CD11b^+^CD11c^+^ dendritic cells (*p* = 0.058, n.s.), and a more pronounced decrease of CD19^+^ B cells (*p* = 0.00284, Kruskal-Wallis test, Figures 4A,B) in stroke mice. In contrast, we found no significant changes in the number of total T cells, CD4^+^ helper T cells, CD8 cytotoxic T cells, TCRγδ T cells, IL-17 producing CD4^+^ cells, IFNγ producing CD4^+^ cells, IL-17 producing TCRγδ^+^ T cells, or IFNγ producing TCRγδ^+^ T cells (Figures 4C,D, Supplemental Figures 4A–F).

### No evidence for overt bacterial translocation after 60 min MCAO

**Figure 5:**
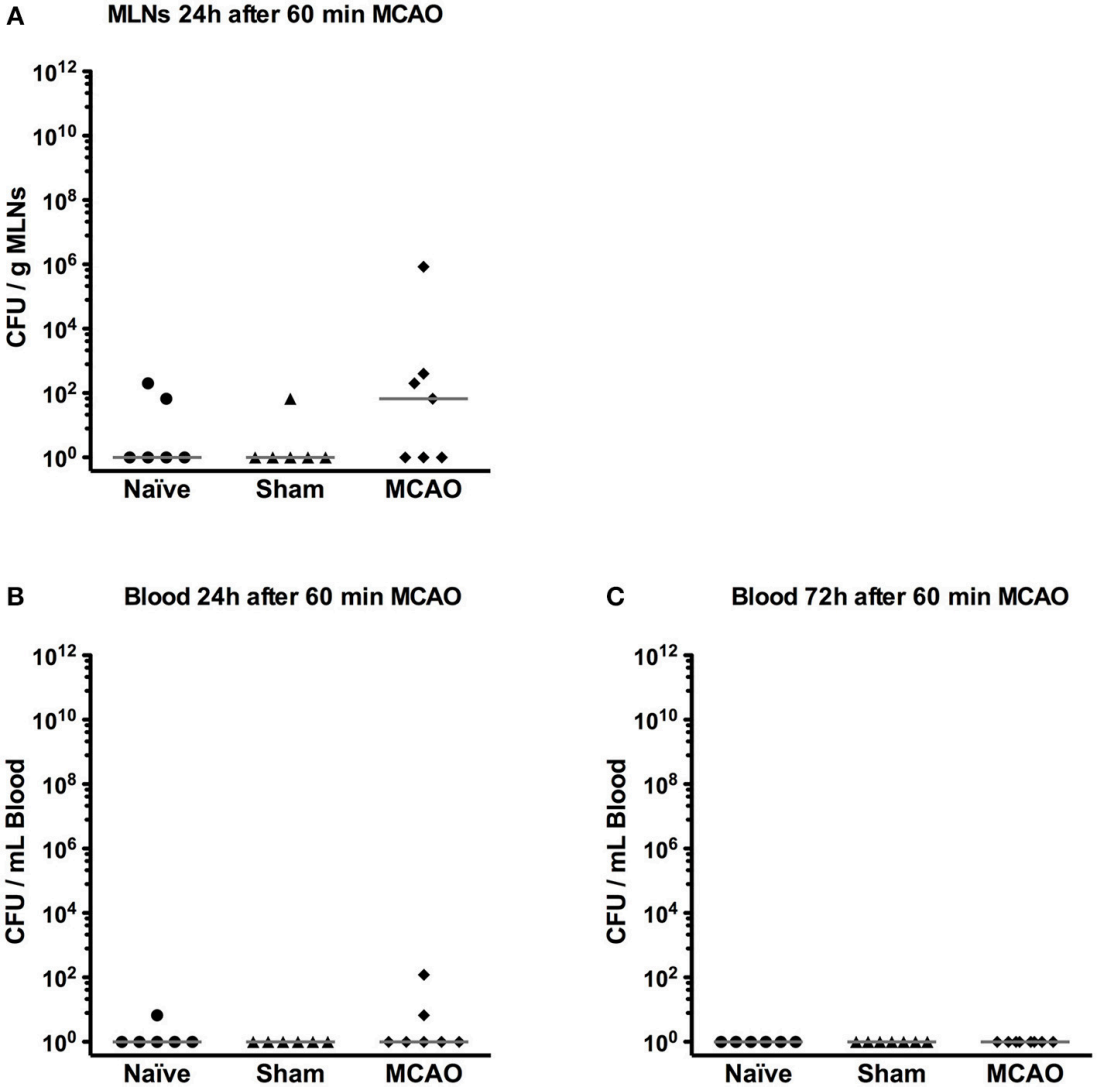
Bacterial translocation was rarely found in mesenteric lymph nodes (MLNs) and blood. **(A)** MLNs were collected and plated on LB agar on day 1. After 24 h incubation, the number of bacterial colonies was counted. **(B)** Blood was sampled and plated on LB agar on day 1 and **(C)** day 3. Bacterial colonies were counted 24 h after plating. All results were presented as colony forming unit (CFU) per gram MLNs or mL blood.

Since in our previous experiments, Gram negative bacteria were the most abundant pathogens in mice developing post-stroke pneumonia (21), we assessed bacterial translocation in MLNs and blood by culturing representative samples on lysogeny broth agar (LB agar) 1 and 3 days after MCAO. Positive cultures in MLNs were observed in 2/6 naïve mice, 1/6 of sham-operated mice and 4/7 of MCAO-operated mice on day 1 (*p* > 0.05, Kruskal-Wallis test), but most of the animals had rather few colonies (< 10^2^ CFU/g MLNs, Figure 5A). In blood, only 1/6 of naïve and 2/7 of MCAO-operated mice showed a few colonies on day 1 and no positive cultures were observed on day 3 (Figures 5B,C).

We next assessed the presence of bacteria and bacterial components in lungs, liver, spleen, MLNs and blood by FISH with a pan-bacterial 16S rRNA gene probe (EUB338). EUB338 probe visualized bacteria in the intestinal lumen, whereas nonsense probe (NON338) did not show cross hybridization (Supplemental Figures 5A,B, images from naïve mice). Bacteria were also observed on the mucosal surfaces of the trachea (Supplemental Figure 5C, naïve mouse). FISH examination revealed a few bacterial signals in peripheral lung tissues (Table 1; Supplemental Figures 5D,E, images from sham-operated animal). In contrast, there were no apparent positive signals in liver, spleen, MLNs and blood on day 1 and 3 (Table 1).

**Table 1:** Bacterial Translocation Ratios.

	**Lung (except trachea)**	**Liver**	**Spleen**	**Blood**	**MLNs**
**DAY 1**					
Naïve	0/6	0/6	0/6	0/6	0/6
Sham	1/6	0/6	0/6	0/6	0/5
MCAO	1/6	0/7	0/7	0/6	0/6
**DAY 3**					
Naïve	1/6	0/6	0/6	0/6	0/6
Sham	0/6	0/6	0/5	0/6	0/5
MCAO	0/7	0/7	0/7	0/7	0/7

*Cerebral ischemia rarely induced bacterial translocation to extraintestinal organs. MLNs indicates mesenteric lymph nodes; MCAO, middle cerebral artery occlusion*.

## Discussion

In light of recent reports underpinning the role of the gut microbiota in stroke and contradictory evidence on intestinal barrier function and bacterial translocation after cerebral ischemia, we performed an exploratory study focusing on gut function after experimental stroke. We specifically addressed the question whether 60 min MCAO in the mouse leads to changes in gut barrier protein expression, intestinal permeability, alterations in the gut associated immune system, or bacterial translocation to extraintestinal organs. We did not find overt changes in these parameters within the first 3 days after middle cerebral artery occlusion. As our exploratory investigation yielded mostly neutral findings we will discuss our results emphasizing the limitation of our approach.

The bacterial community of the intestine, the gut microbiota, was recently identified as an important player in post-stroke pathophysiological events. Until now, two independent studies linked the composition of intestinal microbiota with CNS profile- and brain meninges-infiltrating immune cells, further impacting the lesion size (8, 9). Interestingly, almost 25% of T cells found in the ischemic hemisphere originated from the gut (9). Our group described dramatically worsened outcome after experimental stroke in microbiota-depleted mice without antibiotic protection (22). Data about intestinal function and possible translocation of commensal bacteria from the intestine after stroke are limited until now.

Based on a potential link to the CNS, it is plausible to speculate that intestinal function may be affected by cerebral ischemia (23). Brain injury leads to overactivation of the HPA axis and the autonomic nervous system, with profound effects on the gut and its bacterial communities (9, 24, 25). Additionally, increased activity of these axes leads to systemic immunosuppression, which can disturb immunological barriers, such as in lung and gut, and facilitate development of infectious complications. Physical and immunological barriers are of great importance in defending the body against invading pathogens. Impaired intestinal function, including decreased motility (9) and increased permeability (9, 12, 15) after mouse experimental stroke was already reported by several groups. In contrast to these results we could not find alterations in the intestinal permeability using the lactulose/mannitol/sucralose test. This assay is used as a standard procedure to assess intestinal function in human subjects. Importantly, in our setting, we noted high interindividual variability in this test, particularly in the investigations from day 1–2 after experimental stroke. In previous literature the median percentage excretion of lactulose and mannitol in healthy C57Bl6 animals was 0.57% (0.17–1.55%, 5th to 95th percentile) and 8.99% (2.02–24.29%, 5th to 95th percentile) respectively (26). Accordingly, using small (but common) sample sizes, we would be able to identify only large permeability changes in our experiments. From our results, we conclude that intestinal barrier function does not change dramatically after experimental stroke when measured on day 2 and 3 after injury, while more subtle changes may have been missed. However, when compared to published data, our analysis included investigation of samples collected over 24 h and on later time points after cerebral ischemia (24–48 and 48–72 h after stroke). Previously, the most prominent changes in the permeability using FITC-dextran assay were identified 3 h after cerebral ischemia (12). Additionally, the use of lactulose/mannitol/sucralose assay may be not optimal in the experimental stroke setting, since the results could be possibly affected by decreased food and water intake (affecting renal secretion) related to motor impairment and important when using metabolic cages. This assay, however, provides valuable information about the permeability of small and large intestine separately, does not require sacrificing animals and is not affected by fecal excretion, which can be limited after cerebral ischemia. Definitely, to ultimately answer the question of permeability changes after experimental stroke, detailed investigations using different methods and providing detailed kinetics, would be needed.

The physical barrier in the intestine consists of epithelial tight junctions (TJs) composed of multiple protein subunits. Claudins and occludins (27, 28) appear to be the most relevant ones, as they play a crucial role in forming physical and physiological mucosal barriers in cooperation with intestinal epithelial cells. TJs integrate intestinal physiological, immunological and host-microbial homeostasis by regulating solutes flux along paracellular pathway (27, 28). Disturbances in TJs expression and altered intestinal morphology were already found in traumatic brain injury and experimental stroke (12, 29, 30, 31). In this study, we observed only decreased claudin-1 expression in the ileum 3 days after MCAO, when compared to naïve animals. However, animals on day 3 did not show significant histological and permeability changes in other tests performed.

Numeric and functional changes in leukocyte subsets in spleen, blood and thymus indicating systemic immunosupression after CNS-injury were reported within the first few days after stroke in mice (5, 6, 32). In this study, we found decreased counts of CD11b CD11c+ dendritic cells and B cells in Peyer's patches after cerebral ischemia, when compared to naïve animals. The immunological changes present in sham-operated animals, for instance lower counts of CD11b CD11c+ dendritic cells and B cells (not achieving statistical significance) may be attributed to the effects of surgical intervention, including among others closing the left common carotid artery, tissue damage, psychological stress and anesthesia. B cells predominate in Peyer's patches and differentiate into IgA-producing B cells in the presence of T cells, leading to the elimination of toxins and pathogenic bacteria (33–35). It was already shown that stress before cerebral ischemia induces a significant reduction of IgA in the large intestine and bacterial translocation in a rat stroke model (13). Also, dendritic cells in Peyer's patches are critical for initiating and developing adaptive immune response (34, 36). Hence, stroke-induced reduction of B cells and dendritic cells in Peyer's patches may threaten homeostasis in local and systemic immune system, and contribute to the impaired local antibacterial defense. In line with our results, a previous study from our group had revealed decreased counts of B and T cells in Peyer's patches (PP) of 129SV mice subjected to 60 min of MCAO when compared to sham-operated mice, whereas no significant alterations in intraepithelial and lamina propria lymphocyte subsets were found (37).

Importantly, translocation of bacteria from the intestine to extraintestinal organs after stroke has been observed previously (12–15), and Wong et al. even attributed the origin of stroke-associated pneumonia to this phenomenon (12). This link is, however, highly controversial, since from a clinical point of view dysphagia facilitating aspiration is undoubtedly the most important factor involved in the pathogenesis of stroke-associated pneumonia, and peripheral immunosuppression fosters the development of infection (5, 38). In experimental stroke models bacterial translocation to mesenteric lymph nodes, liver and spleen was observed in rats after permanent MCA occlusion (14), or only in rats subjected to stress before stroke induction (13). Crapser et al. reported translocation to mesenteric lymph nodes at day 3 after cerebral ischemia in young and old mice, whereas only young animals were able to successfully clear translocated bacteria (15). We and others (9) found no evidence for bacterial translocation after cerebral ischemia.

These discrepancies in reported observations may result from stroke severity, using different brain ischemia models, but also from differences in the techniques used to assess the presence or absence of bacterial translocation, including cultivation on microbiological media (13, 14) FISH (9), assessment of translocation after inoculation with *Enterococcus faecalis* (12) or GFP-expressing *E.coli* (15).

All of the above-mentioned methods suffer from several drawbacks. Conventional cultivation has been used as the gold standard procedure and has excellent sensitivity, but detects only viable bacteria, of which only approximately 1% can be cultivated (39) and is prone to sample contamination. Another limitation of cultivation techniques is the need of proper choice of microbiological media. In our investigations, we used only LB agar, since in previous experiments we observed post-MCAO pneumonia of mostly Gram-negative etiology (21).

PCR-derived techniques and FISH methods using 16S or 23S rRNA gene targeted probes are considered as powerful tools for microbial detection (40–42). In PCR, however, it is also well known that false-negative or -positive results can easily occur because of high susceptibility to PCR-inhibitors in tissues and carryover contamination, respectively (43, 44). In contrast, FISH has similar or lower sensitivity to conventional techniques but can visualize most of the bacteria and reveal their localization when tissue sections are used (42, 44). In our study, we were able to visualize intestinal bacteria and bacteria in the airways (Supplemental Figures 5A,C–E) and we did not find apparent bacterial signals in other organs.

When performing inoculation experiments (12) with colonization of the intestine with defined bacteria and assessment of subsequent translocation, it is difficult, if not impossible, to exclude that the lungs may be directly inoculated when oral gavage is performed. It is important to note that MCAO mice, in contrast to sham-operated controls, are prone to bacterial pneumonia after aspirating even very small amounts of microorganisms to the lungs (21). Hence, bacteria found in the lung of orally colonized MCAO mice may originate from the inoculation itself and not from the translocation from the intestine. Moreover, (GFP-expressing) *E.coli* is not found in high numbers in the physiological gut microbiota, and may, therefore, translocate more often than typical commensal bacteria in case of induced colonization and subsequent pathological events. Since mice are coprophagic animals, excreted bacteria may be later found e.g., in the lungs.

Taken together, our exploratory results suggest that neither severe impairment of intestinal barrier nor bacterial translocation is a common event after 60 min MCAO in young adult male mice. Our results will be useful to calculate sample sizes for future confirmatory investigation in this commonly used model of experimental stroke (14). Further studies are required to explore the pathomechanisms involved in the development of stroke-associated infections, the most frequent and the most severe complication of this devastating disorder (1).

## Author contributions

NO, KW, AM, and UD conceived and designed the experiments. NO, KW, PB-K, and TZ performed the experiments. MW performed HPLC for analysis of intestinal permeability. PB-K, CD, and CM contributed to the immunological investigations. OK conducted histological analyses of intestinal samples. NO, KW, PB-K, and CD analyzed the data. AM and UD provided feedback on data analysis. NO, KW, CM, AM, and UD wrote the manuscript.

### Conflict of interest statement

The authors declare that the research was conducted in the absence of any commercial or financial relationships that could be construed as a potential conflict of interest.

## References

[1] Kumar S Selim MH Caplan LR. Medical complications after stroke. Lancet Neurol. (2010) 9:105–18. 10.1016/S1474-4422(09)70266-2 20083041

[2] Rohweder G Ellekjaer H Salvesen O Naalsund E Indredavik B. Functional outcome after common poststroke complications occurring in the first 90 days. Stroke (2015) 46:65–70. 10.1161/STROKEAHA.114.006667 25395415

[3] Hoffmann S Harms H Ulm L Nabavi DG Mackert BM Schmehl I. Stroke-induced immunodepression and dysphagia independently predict stroke-associated pneumonia - The PREDICT study. J Cereb Blood Flow Metab. (2017) 37:3671–82. 10.1177/0271678X16671964 27733675 PMC5718319

[4] Schaller BJ Graf R Jacobs AH. Pathophysiological changes of the gastrointestinal tract in ischemic stroke. Am J Gastroenterol. (2006) 101:1655–65. 10.1111/j.1572-0241.2006.00540.x 16863574

[5] Meisel C Schwab JM Prass K Meisel A Dirnagl U. Central nervous system injury-induced immune deficiency syndrome. Nat Rev Neurosci. (2005) 6:775–86. 10.1038/nrn1765 16163382

[6] Prass K Meisel C Hoflich C Braun J Halle E Wolf T. Stroke-induced immunodeficiency promotes spontaneous bacterial infections and is mediated by sympathetic activation reversal by poststroke T helper cell type 1-like immunostimulation. J Exp Med. (2003) 198:725–36. 10.1084/jem.20021098 12939340 PMC2194193

[7] Chamorro A Meisel A Planas AM Urra X Van De Beek D Veltkamp R. The immunology of acute stroke. Nat Rev Neurol. (2012) 8:401–10. 10.1038/nrneurol.2012.98 22664787

[8] Benakis C Brea D Caballero S Faraco G Moore J Murphy M. Commensal microbiota affects ischemic stroke outcome by regulating intestinal gammadelta T cells. Nat Med. (2016) 22:516–23. 10.1038/nm.4068 27019327 PMC4860105

[9] Singh V Roth S Llovera G Sadler R Garzetti D Stecher B. Microbiota dysbiosis controls the neuroinflammatory response after stroke. J Neurosci. (2016) 36:7428–40. 10.1523/JNEUROSCI.1114-16.2016 27413153 PMC6705544

[10] Singh V Sadler R Heindl S Llovera G Roth S Benakis C. The gut microbiome primes a cerebroprotective immune response after stroke. J Cereb Blood Flow Metab. (2018) 38:1293–8. 10.1177/0271678X18780130 29846130 PMC6092773

[11] Asrat S Davis KM Isberg RR. Modulation of the host innate immune and inflammatory response by translocated bacterial proteins. Cell Microbiol. (2015) 17:785–95. 10.1111/cmi.12445 25850689 PMC4632489

[12] Stanley D Mason LJ Mackin KE Srikhanta YN Lyras D Prakash MD. Translocation and dissemination of commensal bacteria in post-stroke infection. Nat Med. (2016) 22:1277–84. 10.1038/nm.4194 27694934

[13] Caso JR Hurtado O Pereira MP Garcia-Bueno B Menchen L Alou L. Colonic bacterial translocation as a possible factor in stress-worsening experimental stroke outcome. Am J Physiol Regul Integr Comp Physiol. (2009) 296:R979–85. 10.1152/ajpregu.90825.2008 19193944

[14] Tascilar N Irkorucu O Tascilar O Comert F Eroglu O Bahadir B. Bacterial translocation in experimental stroke: what happens to the gut barrier? Bratisl Lek Listy (2010) 111:194–9. 20586145

[15] Crapser J Ritzel R Verma R Venna VR Liu F Chauhan A. Ischemic stroke induces gut permeability and enhances bacterial translocation leading to sepsis in aged mice. Aging (2016) 8:1049–63. 10.18632/aging.100952 27115295 PMC4931853

[16] Hetze S Engel O Romer C Mueller S Dirnagl U Meisel C. Superiority of preventive antibiotic treatment compared with standard treatment of poststroke pneumonia in experimental stroke: a bed to bench approach. J Cereb Blood Flow Metab. (2013) 33:846–54. 10.1038/jcbfm.2013.6 23361393 PMC3677122

[17] Kimmelman J Mogil JS Dirnagl U. Distinguishing between exploratory and confirmatory preclinical research will improve translation. PLoS Biol. (2014) 12:e1001863. 10.1371/journal.pbio.1001863 24844265 PMC4028181

[18] Gelman A.a.L., E. (2013). The garden of forking paths: Why multiple comparisons can be a problem, even when there is no ‘fishing expedition' or ‘p-hacking' and the research hypothesis was posited ahead of time. Available online at: http://www.stat.columbia.edu/g~elman/research/unpublished/p_hacking.pdf

[19] Motulsky HJ Brown RE. Detecting outliers when fitting data with nonlinear regression - a new method based on robust nonlinear regression and the false discovery rate. BMC Bioinformatics (2006) 7:123. 10.1186/1471-2105-7-123 16526949 PMC1472692

[20] Moolenbeek C Ruitenberg EJ. The “Swiss roll”: a simple technique for histological studies of the rodent intestine. Lab Anim. (1981) 15:57–9. 10.1258/002367781780958577 7022018

[21] Prass K Braun JS Dirnagl U Meisel C Meisel A. Stroke propagates bacterial aspiration to pneumonia in a model of cerebral ischemia. Stroke (2006) 37:2607–12. 10.1161/01.STR.0000240409.68739.2b 16946159

[22] Winek K Engel O Koduah P Heimesaat MM Fischer A Bereswill S. Depletion of cultivatable gut microbiota by broad-spectrum antibiotic pretreatment worsens outcome after murine stroke.Stroke (2016) 47:1354–63. 10.1161/STROKEAHA.115.011800 27056982 PMC4839545

[23] Winek K Meisel A Dirnagl U Gut microbiota impact on stroke outcome: Fad or fact? J Cereb Blood Flow Metab. (2016) 36:891–8. 10.1177/0271678X16636890 26945017 PMC4853845

[24] Houlden A Goldrick M Brough D Vizi ES Lenart N Martinecz B. Brain injury induces specific changes in the caecal microbiota of mice via altered autonomic activity and mucoprotein production. Brain Behav Immun. (2016) 57:10–20. 10.1016/j.bbi.2016.04.003 27060191 PMC5021180

[25] Stanley D Moore RJ Wong CHY. An insight into intestinal mucosal microbiota disruption after stroke. Sci Rep. (2018) 8:568. 10.1038/s41598-017-18904-8 29330443 PMC5766598

[26] Volynets V Reichold A Bardos G Rings A Bleich A Bischoff SC. Assessment of the intestinal barrier with five different permeability tests in healthy C57BL/6J and BALB/cJ mice. Dig Dis Sci. (2016) 61:737–46. 10.1007/s10620-015-3935-y 26520109

[27] Turner JR. Intestinal mucosal barrier function in health and disease. Nat Rev Immunol. (2009) 9:799–809. 10.1038/nri2653 19855405

[28] Suzuki T. Regulation of intestinal epithelial permeability by tight junctions. Cell Mol Life Sci. (2013) 70:631–59. 10.1007/s00018-012-1070-x 22782113 PMC11113843

[29] Hang CH Shi JX Li JS Wu W Yin HX. Alterations of intestinal mucosa structure and barrier function following traumatic brain injury in rats. World J Gastroenterol. (2003) 9:2776–81. 10.3748/wjg.v9.i12.2776 14669332 PMC4612051

[30] Feng D Xu W Chen G Hang C Gao H Yin H. Influence of glutamine on intestinal inflammatory response, mucosa structure alterations and apoptosis following traumatic brain injury in rats. J Int Med Res. (2007) 35:644–56. 10.1177/147323000703500509 17900404

[31] Bansal V Costantini T Kroll L Peterson C Loomis W Eliceiri B. Traumatic brain injury and intestinal dysfunction: uncovering the neuro-enteric axis. J Neurotrauma (2009) 26:1353–9. 10.1089/neu.2008.0858 19344293 PMC2989839

[32] Mcculloch L Smith CJ Mccoll BW Adrenergic-mediated loss of splenic marginal zone B cells contributes to infection susceptibility after stroke. Nat Commun. (2017) 8:15051 10.1038/ncomms15051 28422126 PMC5399306

[33] Suzuki K Fagarasan S. How host-bacterial interactions lead to IgA synthesis in the gut. Trends Immunol. (2008) 29:523–31. 10.1016/j.it.2008.08.001 18838301

[34] Jung C Hugot JP Barreau F. Peyer's patches: the immune sensors of the intestine. Int J Inflam. (2010) 2010:823710. 10.4061/2010/823710 21188221 PMC3004000

[35] Suzuki K Nakajima A. New aspects of IgA synthesis in the gut. Int Immunol. (2014) 26:489–94. 10.1093/intimm/dxu059 24872116

[36] Lelouard H Henri S De Bovis B Mugnier B Chollat-Namy A Malissen B. Pathogenic bacteria and dead cells are internalized by a unique subset of Peyer's patch dendritic cells that express lysozyme. Gastroenterology (2010) 138:173–84 e1–3. 10.1053/j.gastro.2009.09.051 19800337

[37] Schulte-Herbruggen O Quarcoo D Meisel A Meisel C. Differential affection of intestinal immune cell populations after cerebral ischemia in mice. Neuroimmunomodulation (2009) 16:213–8. 10.1159/000205514 19246945

[38] Cohen DL Roffe C Beavan J Blackett B Fairfield CA Hamdy S. Post-stroke dysphagia: A review and design considerations for future trials. Int J Stroke (2016) 11:399–411. 10.1177/1747493016639057 27006423

[39] Vartoukian SR Palmer RM Wade WG. Strategies for culture of 'unculturable' bacteria. FEMS Microbiol Lett. (2010) 309:1–7. 10.1111/j.1574-6968.2010.02000.x 20487025

[40] Ludwig W Schleifer KH. Bacterial phylogeny based on 16S and 23S rRNA sequence analysis. FEMS Microbiol Rev. (1994) 15:155–73. 10.1111/j.1574-6976.1994.tb00132.x 7524576

[41] Clarridge JE III. Impact of 16S rRNA gene sequence analysis for identification of bacteria on clinical microbiology and infectious diseases. Clin Microbiol Rev. (2004) 17**:**840–62. 10.1128/CMR.17.4.840-862.2004 15489351 PMC523561

[42] Gosiewski T Flis A Sroka A Kedzierska A Pietrzyk A Kedzierska J. Comparison of nested, multiplex, qPCR; FISH; SeptiFast and blood culture methods in detection and identification of bacteria and fungi in blood of patients with sepsis. BMC Microbiol. (2014) 14:313. 10.1186/s12866-014-0313-4 25551203 PMC4302608

[43] Philipp S Huemer HP Irschick EU Gassner C. Obstacles of multiplex real-time PCR for bacterial 16S rDNA: primer specifity and DNA decontamination of Taq Polymerase. Transfus Med Hemother. (2010) 37:21–8. 10.1159/000265571 20737013 PMC2914405

[44] Rohde A Hammerl JA Appel B Dieckmann R Al Dahouk S. FISHing for bacteria in food–a promising tool for the reliable detection of pathogenic bacteria? Food Microbiol. (2015) 46:395–407. 10.1016/j.fm.2014.09.002 25475309

