# Supplementary material for: Exploratory Investigation of Intestinal Function and Bacterial Translocation After Focal Cerebral Ischemia in the Mouse
Source: Front Neurol. 2018 Nov 19;9:937. doi: 10.3389/fneur.2018.00937 (PMC6254134; doi:10.3389/fneur.2018.00937)
Supplement: Supplementary file 1 [file Data_Sheet_1.pdf]

SUPPLEMENTAL MATERIAL

Exploratory Investigation of Intestinal Function and Bacterial Translocation after Focal Cerebral Ischemia in the Mouse

Naoki Oyama#[1,2], Katarzyna Winek#*[1,2,3], Priscilla Bäcker-Koduah[1,3], Tian Zhang[1,2], Claudia Dames[4], Martina Werich[5], Olivia Kershaw[6], Christian Meisel[4], Andreas Meisel[1,2,3,7], Ulrich Dirnagl[1,2,3,7,8,9]

#these authors contributed equally

Affiliations:

[1] Charité – Universitätsmedizin, corporate member of Freie Universität Berlin, Humboldt-Universitäts zu Berlin, and Berlin Institute of Health, Department of Experimental Neurology

[2] Charité – Universitätsmedizin, corporate member of Freie Universität Berlin, Humboldt-Universitäts zu Berlin, and Berlin Institute of Health, Center for Stroke Research Berlin

[3,] Charité – Universitätsmedizin, corporate member of Freie Universität Berlin, Humboldt-Universitäts zu Berlin, and Berlin Institute of Health, Neurocure Cluster of Excellence

[4] Charité – Universitätsmedizin, corporate member of Freie Universität Berlin, Humboldt-Universitäts zu Berlin, and Berlin Institute of Health, Institute for Medical Immunology

[5] Charité – Universitätsmedizin, corporate member of Freie Universität Berlin, Humboldt-Universitäts zu Berlin, and Berlin Institute of Health, Medical Department, Division of Hepatology and Gastroenterology

[6] Institute of Veterinary Pathology, Faculty of Veterinary Medicine, Freie Universität Berlin, Germany

[7] Charité – Universitätsmedizin, corporate member of Freie Universität Berlin, Humboldt-Universitäts zu Berlin, and Berlin Institute of Health, Department of Neurology

[8] German Center for Neurodegenerative Diseases (DZNE), partner site Berlin, Germany

[9] QUEST – Center for Transforming Biomedical Research, Berlin Institute of Health (BIH), Berlin, Germany

*Corresponding Author:

Katarzyna Winek, MD/PhD
Department of Experimental Neurology
Charité – Universitätsmedizin Berlin
Charitéplatz 1, 10117 Berlin, Germany
E-mail: katrzyna.winek@charite.de

Supplemental Methods

Transient Focal Cerebral Ischemia

Middle cerebral artery occlusion (MCAO) was performed according to the standard operating procedures from the Department of Experimental Neurology, Charité-Universitätsmedizin Berlin, Germany (Dirnagl and group, 2010). Briefly, each mouse was anesthetized using a combination of 1.5-2% isoflurane in 70% nitric oxide and 30% oxygen, and placed in supine position on a heating plate to maintain the body temperature at 36.5°C ± 0.5°C through the procedure. The left common carotid artery (CCA) was exposed by a midline ventral neck incision, and then the external carotid artery and proximal CCA were occluded by a 5-0 silk suture. The proximal internal carotid artery was loosely occluded with a 5-0 silk suture and a silicon hardener-coated nylon filament (7019PK5Re; Doccoll Corp., Redlands, California USA) was introduced into the CCA. The filament was advanced until the tip occluded the origin of the left middle cerebral artery (MCA) causing ischemic lesion in the territory supplied by MCA. At 60 minutes after MCAO, the filament was withdrawn to allow reperfusion. Sham operations were performed by inserting the filament to shortly occlude the MCA and withdrawn immediately for quick reperfusion.

The exclusion criteria in the surgery groups were: - unsuccessful stroke based on histological or MRI assessment, - major surgical event (death) during surgery for the MCAO group, death/humane endpoint before sample collection at the endpoint and - unexpected brain injury based on histological assessment in the sham group.

Evaluation of Infarct Volume

Histology

Infarct volume was evaluated at the endpoint of each experiment. The brains were removed, snap-frozen in pre-cooled 2-methylbutane on dry ice and cut into six 5-µm-thick sections. Infarct lesions were observed through hematoxylin and eosin (HE) staining. To minimize the errors associated with tissue processing for histological evaluation, infarct volumes were calculated by subtracting the intact area of the ipsilateral hemisphere from the area of the contralateral hemisphere as described previously(Swanson et al., 1990).

Magnetic Resonance Imaging

Images were acquired with a small animal 7 T MRI (Bruker BioSpin, Ettlingen, Germany) using a 20 mm diameter quadrature transmit/receive mouse head volume resonator (RAPID biomedical, Rimpar, Germany). Axial T2 weighted images were acquired using a Rapid Acquisition with Relaxation Enhancement (RARE) sequence (20 contiguous slices, slice thickness 0.5 mm, matrix=256x256, field of view=25.6 mm x 25.6 mm, repetition time=4200 ms, echo time=36 ms, RARE factor=8, 6:43 min).

Histological Assessment of Intestines

Intestinal samples were isolated on the day of the surgery, 3, 5, 24 and 21 thereafter as Swiss-rolls (Moolenbeek and Ruitenberg, 1981), fixed in 4% paraformaldehyde and embedded in paraffin. The samples were cut in 5-µm-thick sections, dewaxed and stained with HE for light microscopy to evaluated intestinal morphology.

Isolation of Intestinal Epithelial Cells and Western Blot

One or 3 days after surgery, 30 mm of terminal ileum and proximal colon were dissected, immediately placed on ice and rinsed thoroughly with ice cold phosphate buffer saline. Each segment of intestines was longitudinally opened to expose the intestinal mucosa and the mucosal layers were collected by scraping with a glass slide. The isolated intestinal epithelial cells were washed in Cell Wash Solution provided by Mem-PER™ Plus Membrane Protein

Extraction Kit (Thermo Scientific, IL, USA) and centrifuged at 200 × g, 4°C for 3 minutes, and only supernatant was removed very carefully. The cells were suspended in Permeabilization Buffer (Mem-PER^{TM} Plus Membrane Protein Extraction Kit) which contains a protease inhibitor cocktail (Complete Mini; Roche, Basel, Switzerland) and a phosphatase inhibitor cocktail (PhosSTOP; Roche), frozen in liquid nitrogen and stored at –80°C until further processing.
To evaluate tight junction proteins expression, cytosolic and membrane proteins were separately extracted from isolated cells according to the manufacturer's instructions (Mem-PER^{TM} Plus Membrane Protein Extraction Kit). The protein concentration was determined using Pierce^{TM} BCA Protein Assay Kit (Thermo Scientific). Protein preparations were diluted 1:1 with sample buffer containing 100mM Tris-HCl, pH 6.8, 4% SDS, 20% glycerol, 12% β-mercaptoethanol and 0.05% bromophenol blue, followed by heating at 95°C for 5 minutes. The protein samples were electrophoresed on 4–20% precise^{TM} protein gels (Thermo Scientific) and the separated proteins were transferred onto polyvinylidene difluoride membranes (Immobilon-P; Millipore, Bedford, MA, USA). The membrane was blocked with 5% skim milk or 3% bovine serum albumin (fraction V) in Tris-buffered saline (pH 7.6) with 0.1% Tween-20 (TBS-T) for 60 minutes and then incubated with the primary antibodies overnight at 4°C. The membranes were washed in TBS-T and incubated with horseradish peroxidase-conjugated anti-mouse IgG (1:100000) or anti-rabbit IgG (1:5000; GE Healthcare, Buckinghamshire, UK) for 2 hours at room temperature. The blots were then developed using Super Signal West Dura Extended Duration Substrate (Thermo Scientific). The chemiluminescent signals were detected by a cooled-CCD camera (Proline 9000; Finger Lakes Instrumentation, Lima, NY, USA) and quantified using ImageJ 1.46r software (National Institute of Health, Bethesda, MD, USA). The following primary antibodies were used: mouse anti-claudin-1 monoclonal antibody (1:500; Life Technologies, Grand Island, NY, USA), rabbit anti-occludin polyclonal antibody (1:250; Life Technologies) and rabbit anti-β-actin polyclonal antibody (1:5000; Cell signaling Technology, Beverly, MA, USA).

Intestinal Permeability Assessment (Mannitol/Lactulose/Sucralose Test)
To evaluate intestinal permeability, a sugar-absorption test was performed. Mannitol, a monosaccharide, is passively absorbed through the transcellular pathway and considered as a biological marker of transcellular permeability. In contrast, lactulose and sucralose, disaccharides, can be poorly absorbed through the paracellular pathway. The key molecules to decide the permeability of lactulose and sucralose are tight junction proteins. Therefore, they are considered as markers of paracellular permeability. Lactulose can be metabolized in large intestine and is used to evaluate the paracellular permeability of small intestine. Sucralose can't be metabolized in intestine and is used to assess the paracellular permeability of whole intestine. Mice were habituated in metabolic cages for 1 hour per day, 3 times before the experiment. On day 0 or 1 after surgery, mice were fasted overnight (for 8 hours). On day 1 or 2, each mouse was given 0.25 ml of a solution containing mannitol (8 mg/mL), lactulose (12 mg) and sucralose (6mg) by gavage and placed on a metabolic cage. One hour later, the mice regained access to food and water *ad libitum*. To provide sufficient fluid supply, all animals were additionally injected with 0.5 ml 0,9% NaCl. Absorbed sugars are fully excreted in urine within 6 hours, so the urine was collected 24 hours after sugar administration, and the concentrations of the sugars were measured using high-performance liquid chromatography (HPLC)(Buhner et al., 2006). Percent excretion in urine was expressed as a percentage of the dose ingested [% urinary excretion = 100 × urinary concentration of sugar (mg/mL) × urine volume (mL)/total ingested (mg)].

Flow Cytometry Analysis in Peyer's Patches

The whole intestine was dissected on day 1 and all Peyer's patches were removed. Single cell suspensions from Peyer's patches were prepared by forcing the tissues through a fine wire mesh. To obtain single cell suspensions from Peyer's patches, the patches were initially digested 30 minutes at 37°C in RPMI containing 10% fetal calf serum (FCS; Biochrom, Berlin, Germany) and 3.5 mg collagenase A (Roche, Basel, Switzerland). Cells were washed, resuspended in RPMI 1640 medium containing penicillin, streptomycin, 2 mM glutamine, 10% FCS (Biochrom, Berlin, Germany) and 2×10^6 cells were stimulated for 4 hours with 25 ng/ml phorbol 12-myristate-13-acetate (PMA) and 1 µg/ml ionomycin. Cytokine secretion was inhibited by addition of 5 µg/ml Brefeldin A after the first hour. For flow cytometric analysis, the following fluorescently labeled anti–mouse monoclonal antibodies (BD Bioscience, BioLegend or eBioscience, Heidelberg, Germany) were used: CD19 (6D5), CD3 (145-2C11), CD4 (RM4-5), CD8 (53-6.7), TCRγδ (GL3), CD11b (M1/70), CD11c (N418), IFNγ (XMG1.2) and IL-17 (TC11-18H10.1). Dead cells were excluded by using LIVE/DEAD Fixable Aqua (Invitrogen, Waltham Massachusets, USA). Cell phenotyping was performed on LSRFortessa flow cytometer using FACSDiva software (BD Biosciences, Heidelberg, Germany). Data were analyzed using FlowJo software (Tree Star Inc., Ashland Oregon, USA). Analysis was performed using the following combination of lineage markers: T cells ($CD11b^-CD3^+CD19^-$), T helper cells ($CD11b^-CD19^-CD3^+CD4^+$), cytotoxic T cells ($CD11b^-CD19^-CD3^+CD8^+$), B cells ($CD11b^-CD3^-C19^+$), γδT cells ($CD11b^-CD19^-CD3^+TCR\gamma\delta^+$), and myeloid dendritic cells ($CD11b^+CD11c^+$).

Assessment of Bacterial Translocation (Microbiological Investigation)
Mesenteric lymph nodes (MLNs) were dissected on day 1 and placed with sterile phosphate buffer saline onto a 100µm cell strainer (BD Falcon; BD Bioscience, Bedford, MA, USA) inside a 60 mm Petri dish. The tissue was smashed and pushed through the strainer using the plunger of a 5 mL syringe to obtain tissue homogenates. Blood was collected from inferior vena cava with heparin-rinsed 1mL syringe and 23 gauge needle. Three different dilutions (1:1, 1:100 and 1:1000) were prepared from MLNs homogenates and blood samples. Fifty µL of each dilution was plated on LB agar, incubated with 5% CO_2 at 37°C for 24 hours, and then all bacterial colonies were counted. The bacterial load in the samples was recalculated based on the number of colony forming units (CFU) on plates from the dilutions.

Assessment of Bacterial Translocation (Fluorescence In Situ Hybridization: FISH)
A FISH method was used to visualize not only viable bacteria but also dead bacteria and structural components of bacteria in extraintestinal organs as described previously(Swidsinski et al., 2005). Lung, liver, spleen, MLNs and blood were isolated or collected 1 and 3 days after surgery. Except for blood and MLNs homogenates, the tissues were fixed in modified Carnoy's solution (absolute ethanol: glacial acetic acid: chloroform = 6: 6: 1 by volume) and embedded in paraffin using standard techniques. Paraffin blocks were cut into 5-µm-thick sections (8-10 sections per tissue) and placed on SuperFrost slides (R. Langenbrinck, Emmendingen, Germany). For blood and MLNs homogenates, 5 µL of samples were used to make a blood/MLNs smear on a glass slide, dried and fixed in modified Carnoy's solution.

For the detection of all bacteria in tissue, fluorescence in situ hybridization (FISH) using oligonucleotide probes targeting bacterial 16S rRNA gene, which has been highly conserved between different species of bacteria, was employed. As a pan-bacterial probe, 5'-end Alexa Fluor (AF) 488-conjugated oligonucleotide probe (EUB338: 5'-GCTGCCTCCCGTAGGAGT-3', Eurofins MWG Operon, Ebersberg, Germany) was used for the current study. To assess non-specific binding, we also employed AF 488-labeled nonsense probe (NON338: 5'-ACTCCTACGGGAGGCAGC-3', Eurofins MWG Operon)(Amann et al., 1990). The sections were dewaxed and permeabilized with lysozyme

(Sigma-Aldrich, St. Louis, MO, USA) solution (1 mg/mL lysozyme, 100mM Tris-HCl, 50mM EDTA, pH8.0) for 30 minutes at 37°C and then incubated in pre-warmed hybridization solution (30% formamide, 20 mM Tris-HCl, 0.9 M NaCl, 0.01% SDS; pH 7.4) containing 1 μM oligonucleotide probes at 46°C overnight. The sections were rinsed in 2 × saline sodium citrate and stained with 2 μg/mL DAPI (Sigma-Aldrich) solution for 3 minutes. FISH and DAPI signals were observed under a Leica DMI3000 B fluorescence microscope (Leica Microsystems GmbH, Wetzlar, Germany) and bacterial signals in tissues were counted.

Statistics

Data are expressed as the mean ± standard deviation and dot plots. Differences between two means were examined by the Mann-Whitney U test in permeability test (experiment 2-1) and bacterial translocation data. Kruskal-Wallis test with Dunn's correction was used for multiple comparisons in Western blot, permeability test (experiment 2-2), immunological and bacterial translocation data. Statistics was performed using GraphPad Prism software version 5.01 (GraphPad Software, La Jolla, CA, USA).

Supplemental Figures and Table

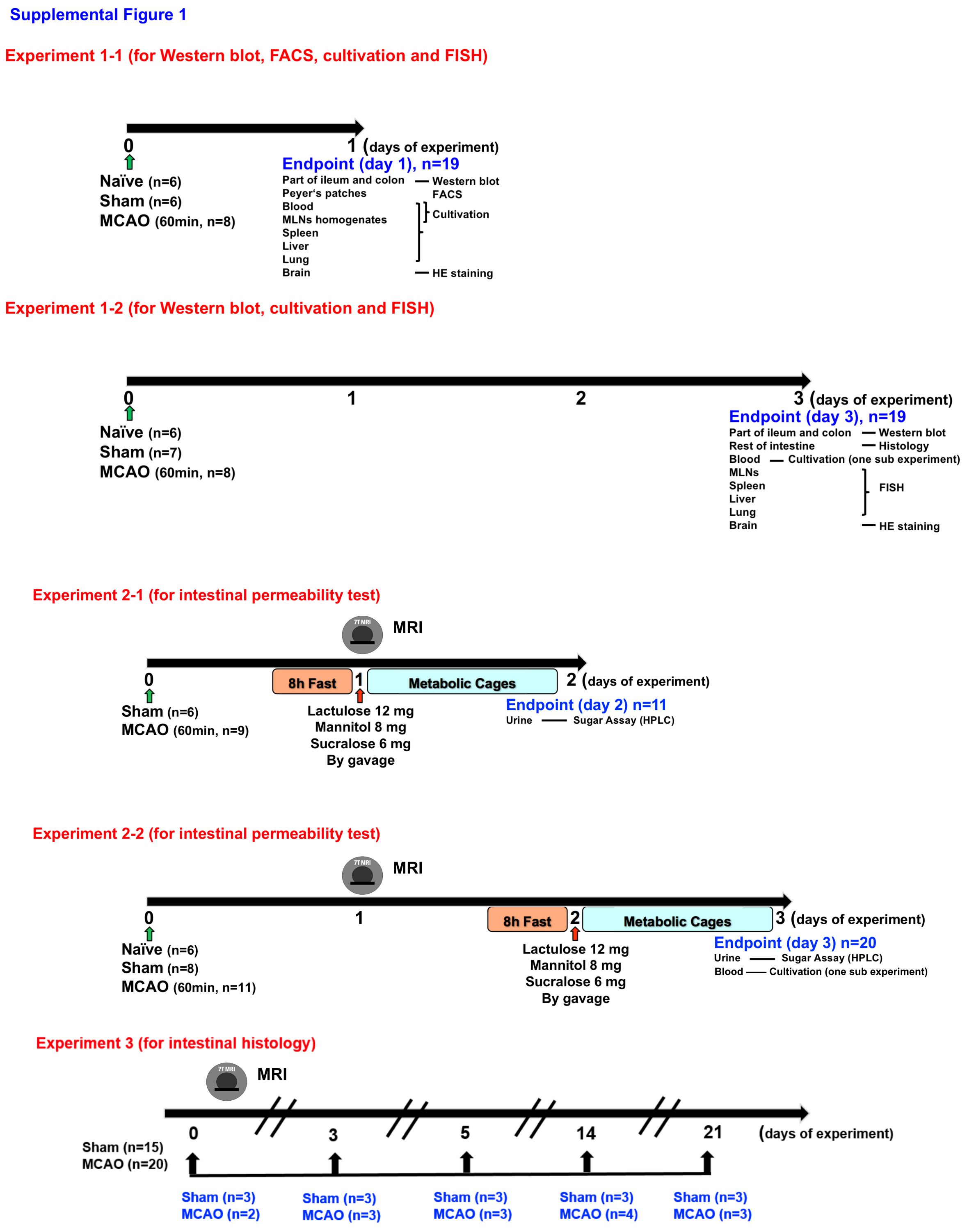

Supplemental Figure 1: Experimental Design

In the experiments 1-1 and 1-2 (for these setups results were pooled from two experimental runs), mice were sacrificed on day 1 or 3 and the tissues were collected for the following assessment: Western blot, flow cytometry (FACS) analysis, cultivation and fluorescence in situ hybridization (FISH). In the experiments 2-1 and 2-2, each mouse was fasted for 8 hours, was given a sugar solution, and then was placed on a metabolic cage on day 1 or 2. Twenty-four hours later, urine was collected for the measurement of sugars concentration. In the

experiment 3, intestines from the mice were collected on day 0, 3, 5, 14 and 21 after MCAO or sham operation for hematoxylin and eosin staining. MCAO indicates middle cerebral artery occlusion; MLNs, mesenteric lymph nodes.

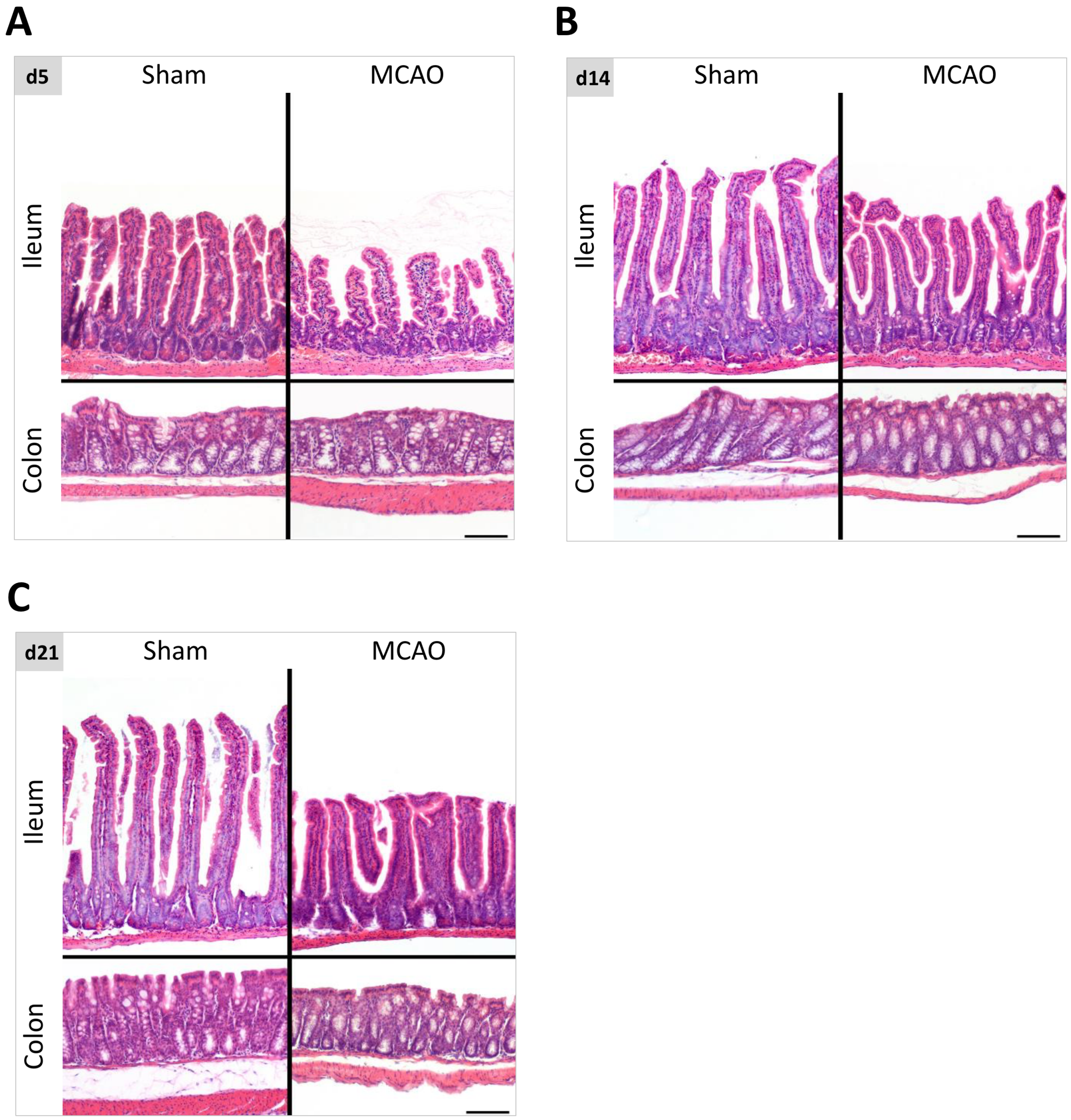

Supplemental Figure 2: Histological pictures of ileum and colon 5, 14 and 21 days after MCAO/sham surgery.

Similar to earlier time points, found no apparent evidence of disarrangement, erosion, ulceration and inflammation in intestinal submucosa and mucosa including epithelial cells and lamina propria, scale bar = 100 µm

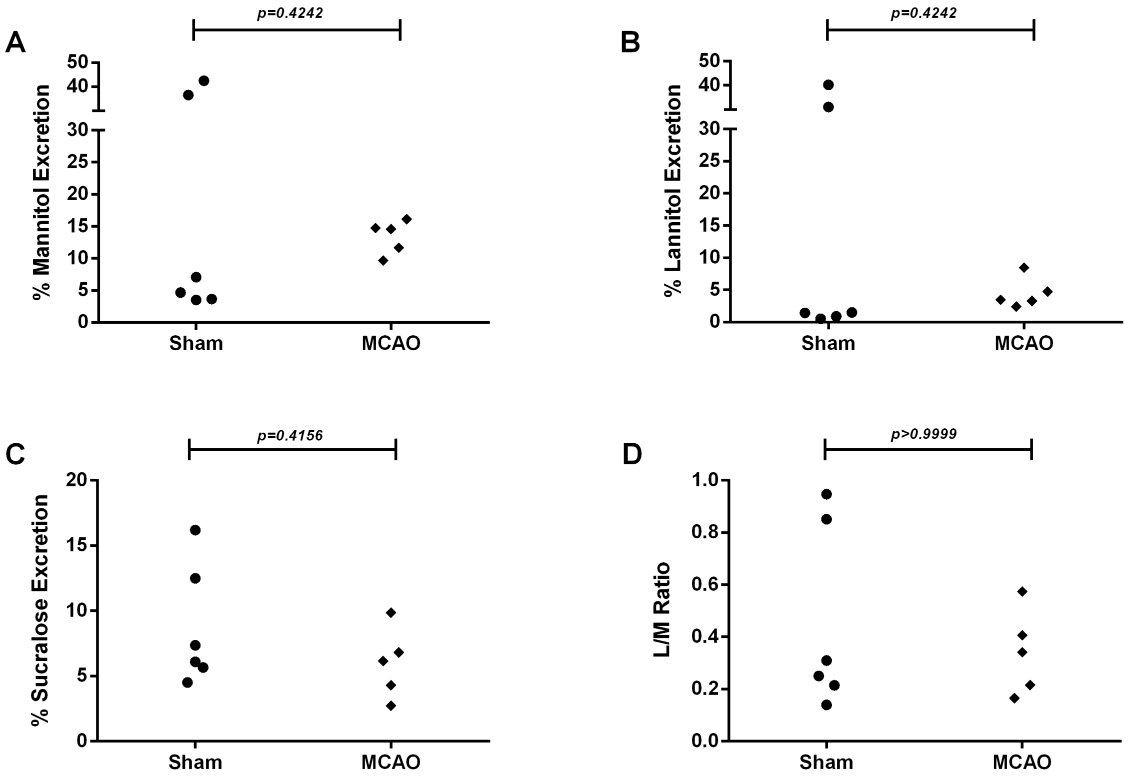

Supplemental Figure 3: Urinary Sugar Concentration (Intestinal Permeability Test)

Ischemia did not change measurably intestinal permeability on day 1–2 assessment. **A**, Percent urinary excretion of mannitol, (**B**) lactulose, (**C**) and sucralose. **D**, Excretion ratio of lactulose and mannitol (L/M ratio). Percent excretion in urine = 100 × urinary concentration of sugar (mg/mL) × urine volume (mL)/total ingested (mg). The groups were compared using Mann-Whitney U test. MCAO indicates middle cerebral artery occlusion.

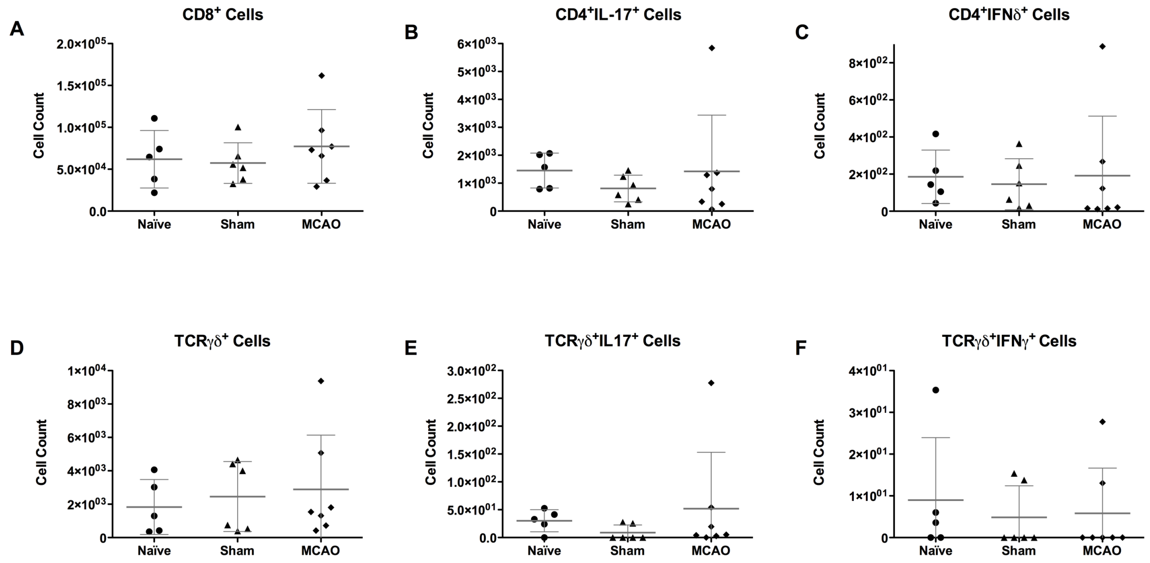

Supplemental Figure 4: Immune Cell Populations in Peyer's Patches

Ischemia did not change significantly following lymphocyte subpopulations: (**A**) CD8+ cells (cytotoxic T cells), (**B**) $CD4^{+}IL\text{-}17^{+}$ cells (IL-17 producing helper T cells), (**C**) $CD4^{+}IFN\gamma^{+}$ cells (IFNγ producing helper T cells), (**D**) $TCR\gamma\delta^{+}$ T cells, (**E**) $TCR\gamma\delta^{+}IL\text{-}17^{+}$ cells (IL-17 producing $TCR\gamma\delta^{+}$ T cells), and (**F**) $TCR\gamma\delta^{+}$ $IFN\gamma^{+}$ cells ($IFN\gamma^{+}$ producing $TCR\gamma\delta^{+}$ T cells). MCAO indicates middle cerebral artery occlusion. Data are expressed as the mean ± standard deviation. The groups were compared using Kruskal-Wallis test with Dunn's post-hoc ($p>0.05$).

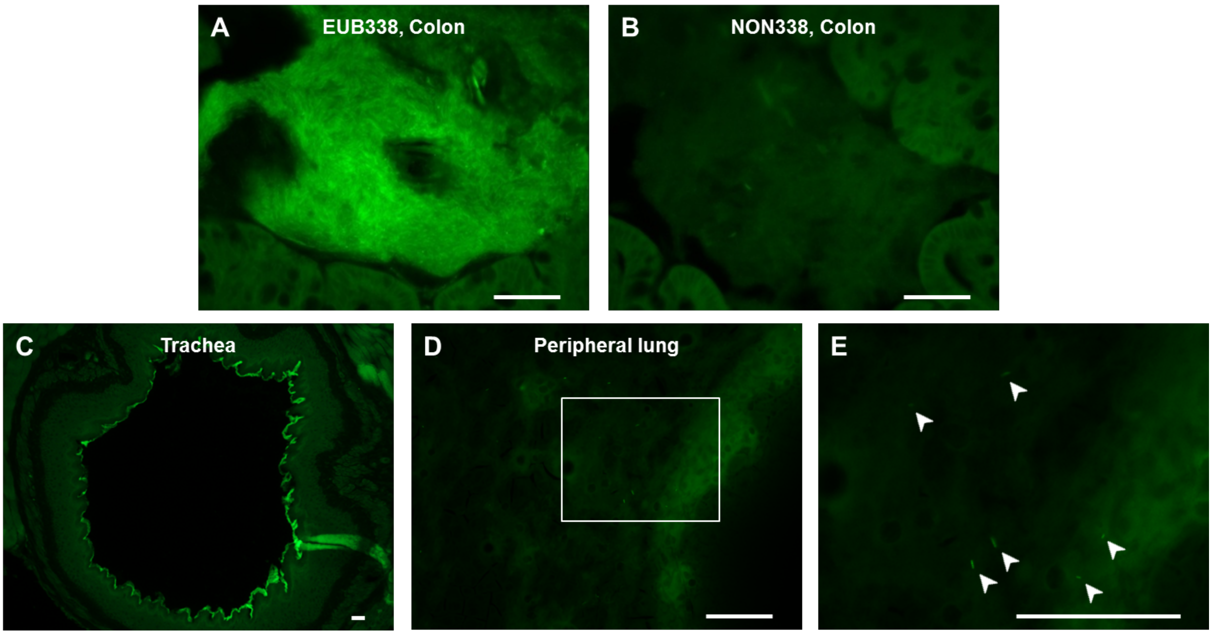

Supplemental Figure 5: Assessment of Bacterial Translocation With Fluorescence in Situ Hybridization (FISH)

A. A pan-bacterial 16S rRNA gene probe, EUB338 visualized commensal bacteria in colon as bright green signals. **B.** A nonsense probe, NON338 did not recognize bacteria in colon. **C**. Lung commensal bacteria along the surface of trachea. (A, B, C show representative images from a naïve mouse). **D.** A few bacterial signals in peripheral lung. **E,** Higher magnification of the boxed region (images from a sham-operated animal). Arrowheads indicate bacteria in tissues. Scale bar = 50 µm.

Experiment	Endpoint	Number of mice entering the study	Excluded animals	Reason of exclusion	Group
1-1	1d	10	1	died on day 0	MCAO
	1d	10	no		
1-2	3d	11	1	brain infarction in histology	Sham
			1	humane endpoint/death	MCAO
	3d	10	no		
2-1	1d	15	4	humane endpoint/death	MCAO
2-2	3d	25	1	died on day 0	sham
			4	humane endpoint/death	MCAO
3	0d - 21d	35	1	no lesion in MRI	MCAO
			4	humane endpoint/death	MCAO

Supplemental Table 1: List of excluded animals

Following general exclusion criteria was implemented in this study: no signs of infarction in histological/MRI investigation in the MCAO group, death on the day of surgery, detectable brain lesions in the sham group, mortality before reaching the endpoint.

Additionally, in the specific analyses samples were excluded only in case of technical problems: Western blot (control ß-actin signal compromised), ileum 1 sample from day 1 and 3 samples for day 3; permeability – no sucralose measurement was performed in 1 animal from the naïve group and 1 sham mouse; FACS – 1 sample from a naive animal; FISH –lung, blood and MLN samples from 1 MCAO mouse in investigation on day 1 and spleen and MLN sample from a sham-operated mouse in the investigation from day 3.

References

Amann, R.I., Binder, B.J., Olson, R.J., Chisholm, S.W., Devereux, R., and Stahl, D.A. (1990). Combination of 16S rRNA-targeted oligonucleotide probes with flow cytometry for analyzing mixed microbial populations. *Appl Environ Microbiol* 56**,** 1919-1925.

Buhner, S., Buning, C., Genschel, J., Kling, K., Herrmann, D., Dignass, A., et al. (2006). Genetic basis for increased intestinal permeability in families with Crohn's disease: role of CARD15 3020insC mutation? *Gut* 55**,** 342-347.

Dirnagl, U. et al (2010). Standard operating procedures (SOP) in experimental stroke research: SOP for middle cerebral artery occlusion in the mouse. *Nature Precedings (Available online* <http://precedings.nature.com/documents/3492/version/2>*).*

Moolenbeek, C., and Ruitenberg, E.J. (1981). The "Swiss roll": a simple technique for histological studies of the rodent intestine. *Lab Anim* 15**,** 57-59.

Swanson, R.A., Morton, M.T., Tsao-Wu, G., Savalos, R.A., Davidson, C., and Sharp, F.R. (1990). A semiautomated method for measuring brain infarct volume. *J Cereb Blood Flow Metab* 10**,** 290-293.

Swidsinski, A., Loening-Baucke, V., Lochs, H., and Hale, L.P. (2005). Spatial organization of bacterial flora in normal and inflamed intestine: a fluorescence in situ hybridization study in mice. *World J Gastroenterol* 11**,** 1131-1140.
